# d-glutamate and Gut Microbiota in Alzheimer’s Disease

**DOI:** 10.3390/ijms21082676

**Published:** 2020-04-11

**Authors:** Chun-Hung Chang, Chieh-Hsin Lin, Hsien-Yuan Lane

**Affiliations:** 1 Institute of Clinical Medical Science, China Medical University, Taichung 40402, Taiwan; chang763@gmail.com; 2 Department of Psychiatry & Brain Disease Research Center, China Medical University Hospital, Taichung 40402, Taiwan; 3 An Nan Hospital, China Medical University, Tainan 709025, Taiwan; 4 Graduate Institute of Biomedical Sciences, China Medical University, Taichung 40402, Taiwan; 5 Kaohsiung Chang Gung Memorial Hospital, Chang Gung University College of Medicine, Kaohsiung 83301, Taiwan; 6 School of Medicine, Chang Gung University, Taoyuan 33302, Taiwan; 7 Department of Psychology, College of Medical and Health Sciences, Asia University, Taichung 41354, Taiwan

**Keywords:** Glutamate, gut microbiota, dementia, brain–gut–microbiota axis, Alzheimer’s disease, *N*-methyl-d-aspartate glutamate receptor

## Abstract

Background: An increasing number of studies have shown that the brain–gut–microbiota axis may significantly contribute to Alzheimer’s disease (AD) pathogenesis. Moreover, impaired memory and learning involve the dysfunction neurotransmission of glutamate, the agonist of the *N*-methyl-d-aspartate receptor and a major excitatory neurotransmitter in the brain. This systematic review aimed to summarize the current cutting-edge research on the gut microbiota and glutamate alterations associated with dementia. Methods: PubMed, the Cochrane Collaboration Central Register of Controlled Clinical Trials, and Cochrane Systematic Reviews were reviewed for all studies on glutamate and gut microbiota in dementia published up until Feb 2020. Results: Several pilot studies have reported alterations of gut microbiota and metabolites in AD patients and other forms of dementia. Gut microbiota including *Bacteroides vulgatus* and *Campylobacter jejuni* affect glutamate metabolism and decrease the glutamate metabolite 2-keto-glutaramic acid. Meanwhile, gut bacteria with glutamate racemase including *Corynebacterium glutamicum, Brevibacterium lactofermentum*, and *Brevibacterium avium* can convert l-glutamate to d-glutamate. N-methyl-d-aspartate glutamate receptor (NMDAR)-enhancing agents have been found to potentially improve cognition in AD or Parkinson’s disease patients. These findings suggest that d-glutamate (d-form glutamate) metabolized by the gut bacteria may influence the glutamate NMDAR and cognitive function in dementia patients. Conclusions: Gut microbiota and glutamate are potential novel interventions to be developed for dementia. Exploring comprehensive cognitive functions in animal and human trials with glutamate-related NMDAR enhancers are warranted to examine d-glutamate signaling efficacy in gut microbiota in patients with AD and other neurodegenerative dementias.

## 1. Introduction

Alzheimer’s disease (AD), the most common cause of dementia, is a progressive, incurable neurodegenerative disease affecting memory and other cognitive functions that interfere with life functions. Acetylcholinesterase inhibitors (AChEI) and memantine (*N*-methyl-d-aspartate (NMDA) receptor antagonist) presently are the main therapeutic drugs for AD [1]. Memantine, an uncompetitive NMDAR partial antagonist, has been developed on the basis of the hypothesis of NMDAR overactivation in moderate–severe AD [2]. However, it shows limited efficacy in mild cognitive impairment (MCI) and mild AD [3]. Moreover, NMDAR antagonists like Ketamine may impair spatial learning and verbal information ability [4]. On the other hand, a pilot study has found NMDAR enhancers such as sodium benzoate may improve cognitive and overall functions in patients with early-phase AD [5].

Glutamate, the agonist of NMDAR and a major excitatory neurotransmitter in the mammalian central nervous system (CNS) [6], is intracellularly located and extensively distributed in the brain. Its concentration in the wet tissue is 5–15 μmol/g [7,8], while its concentration in the synaptic cleft at resting conditions is approximately 0.6 μM [9]. During synaptic transmission, glutamate concentration can increase above 10 μM at spatially localized extracellular regions [10]. Glutamate can be synthesized by several metabolic pathways [11], and removed by the glutamate uptake/transporter system [12]. Glutamate and its receptors, mainly ligand-gated ionotropic glutamate receptors (iGluRs), mediate the vast majority of excitatory neurotransmissions in the mammalian CNS. The receptors also play fundamental roles in synaptic plasticity, the underlying molecular mechanism of learning and memory [13]. Their crucial roles in excitatory neurotransmission indicate that normal signaling disruption via iGluRs is implicated in a wide range of neuropathological disorders and diseases including epilepsy, brain damage, AD, Parkinson’s disease, Huntington’s disease, multiple sclerosis, and schizophrenia, making iGluRs important drug targets for the therapeutic purposes [14,15].

Molecular chirality has been found to affect protein folding, neuronal proliferation, and brain functional laterality and plays an important role in cognition, behavior, and psychiatry [16]. Previous studies reported enantiomers may differ from one another markedly because of different pharmacodynamics and pharmacokinetic properties [17]. A recent research finding supports that d-type amino acids are novel neurotransmitters [18]. However, studies on d-glutamate are limited and their role in neurocognitive function remains unclear. Animal studies have reported higher d-glutamate levels in specific brain regions [19,20], and a pilot human study showed that d-glutamate levels are associated with cognitive functions in AD or MCI patients [21].

The gut microbiota is a complex ecosystem in the human gastrointestinal (GI) tract. The gene set of gut microbiota is approximately 150 times larger (estimated about 4 × 106 genes) than the human gene set (estimated about 26,600 genes) [22,23,24]. Over 99% of GI tract microbiota are anaerobic bacteria and they play important roles in physiological homeostasis and metabolism functions including pathogen displacement, immune system development, barrier fortification, vitamin K synthesis, and nutrient absorption; however, this is often referred to as the “forgotten organ” [25]. Moreover, they are found to involve in the microbiota–gut–brain axis bidirectionally connecting neural [26], immune [27], endocrine [28] and metabolic pathways [29,30]. Recent studies suggest that the gut microbiota play a critical role in neurodegenerative diseases including AD or various types of dementia [31,32]. Pilot studies reported that glutamate metabolized by gut microbiota can be linked to obesity [33], seizure [34], autism [35] and cognition [36,37].

This review’s purpose is to discuss the involvement of glutamatergic neurotransmission and gut microbiota in AD, focusing on the contribution of NMDAR signaling in AD.

## 2. Methods

### Search Strategy

PubMed, Cochrane Systematic Reviews, and Cochrane Collaboration Central Register of Controlled Clinical Trials databases were searched for studies on glutamate and gut microbiota in dementia, from the earliest record to Feb 2020. Review studies that investigated glutamate for dementia or AD patients were analyzed, and included trials and related review articles were manually reviewed for relevant references. Literature not written in English or unavailable in full-text form were not included. The search strings used are the following: “glutamate AND (dementia OR Alzheimer’s disease),” “gut microbiota AND (dementia OR Alzheimer’s disease),” and “glutamate AND gut microbiota.” This article reviews and summarizes these clinical trials.

## 3. d-glutamate from Food

In addition to l-form amino acids, in recent years, d-form amino acids have been found to exist in coffee, cheese, fish, vegetables, fruits, alcoholic beverages, vinegar, ham/meat, milk and milk powder [38]. Table 1 summarized the reported percentage of d-glutamate relative to the total amino acid amounts. High levels of d-glutamate are represented in fermented foods. The relative amount of d-glutamate is 32%–41% in roasted coffee and 27.4% in instant coffee [39,40]. The relative amount of d-glutamate is 24.2% in Yakult, 15% in Parmigiano Reggiano, and 12.4% in yogurt [41,42,43]. Different fermentation processes may contribute different formation of d-amino acids. Lactic fermentation with lactic acid bacteria produces various kinds of D-amino acids [44]. For example, Lactobacillus plantarum glutamate racemase converts l-glutamate into d-glutamate [45].

## 4. Pathways of d-glutamate Metabolism in Mammals

Glutamate metabolism in humans has been well researched in previous studies. After intaking foods containing glutamate, in the gastrointestinal tract, selective transporters like excitatory amino acid carrier C1 (EAAC1) on the apical membrane of enterocytes can absorb the most amino acids [46]. During the first-pass effect, approximately 75%–96% of enteral glutamate is transferred to the splanchnic vessels. Moreover, >80% of this glutamate is converted to energy supporting intestinal function [47,48]. Glutamate may also be involved in gut protein synthesis. They are converted into carbon and nitrogen donors and involved in several metabolic pathways including synthesis of essential amino acids such as arginine and proline, citrulline, and the protective molecule glutathione [49].

**Figure 1:**
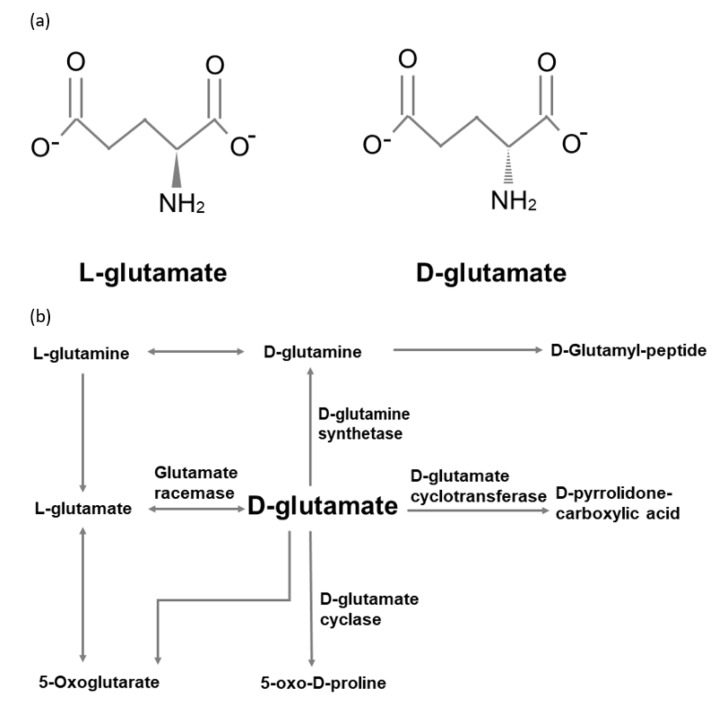
(**a**) l-glutamate and d-glutamate. (**b**) d-glutamate metabolic pathways.

However, the metabolic pathways of d-glutamate in humans remain unclear. Compared with other amino acids, d-glutamate cannot be oxidized by the d-amino acid oxidases (DAOs) [50]. Raj and his colleagues enrolled four healthy volunteers to intake 2g d-glutamate [51]. They monitored the plasma d-glutamate level and urine excretion over a three-hour postload period. They found that plasma d-glutamate level increased 10-fold in the first hour and then reached a plateau over the remaining time course. On the other hand, both plasma level and urine level of d-pyrrolidone carboxylic acid were noted to be higher than d-glutamate. Their results imply that d-glutamate may be transported into cells and metabolized into d-pyrrolidone carboxylic acid. Moreover, d-glutamate cyclase has been found in heart mitochondria to convert d-glutamate into 5-oxo-d-proline [52]. Figure 1 summarizes the d-glutamate metabolic pathways modified from the Kyoto Encyclopedia of Genes and Genomes database.

## 5. Transportation through the Blood–Brain Barrier

The glutamate concentration in the systemic circulation is lower (10–50 µM) than in other amino acids. This may prevent harm to sensitive regions like the brain brought about by increased levels of glutamate [53]. Animal studies using immunohistochemical methods have found a higher level of d-glutamate in the specific brain regions including the subparafascicular thalamic nucleus, the superior colliculus, the mesencephalic central grey matter, and in the dorsal raphe nucleus [54].

The blood–brain barrier (BBB), the endothelial lining of the brain capillaries, regulates the transportation of glutamate. Brain capillary endothelial cells can mediate brain-to-blood glutamate efflux [55,56]. Glutamate concentrations are 5–50-fold lower in the blood, and this concentration gradient prevents glutamate entry to the brain [57]. The two membranes of the BBB include luminal (blood facing) and abluminal (brain facing) membranes which work in a complementary fashion. Facilitative transport of glutamate exists only on the luminal membranes, while Na^+^-dependent transport systems for glutamate exists on the abluminal membrane. Glutamate transporters are transmembrane proteins which are translated from the solute carrier (SLC) gene families such as SCL 1, SLC 7, SLC 17, and SLC25 [58]. The SLC 1 family includes seven members. Five of them are excitatory amino acid transporters (ETTAs) which can transport glutamate [59]. EAAT4 has the highest affinity for transporting glutamate while EAAT5 has the lowest affinity [60]. Through EAATs, glutamate is actively removed from the synaptic cleft and then transported into the cytosol. According to previous findings, EAATs include EAAT1/GLAST, EAAT2/GLT-1, EAAT3/EAAC1, EAAT4, and EAAT5 [61,62]. EAAT1-3 are the main transporters of intraparenchymal glutamate [58]. EAAT4 is located in the Purkinje cells in the cerebellum, while EAAT5 is localized in the retina. GLAST and GLT-1 are mostly expressed by astrocytes. EAAC1 is mainly located in postsynaptic neurons. Astrocytic cytosol contains high levels of glutamine synthase, transforming glutamate into glutamine. After glutamine is formed, it is transported into the extracellular fluid. Neurons uptake glutamine and convert glutamine into glutamate by the deaminase [60]. This transportation and metabolization prevents the high levels of extracellular glutamate resulting in neuron excitotoxicity [63]. However, the different anatomical distribution and transportation of d-glutamate in humans remains unclear. Further studies are needed to investigate the specific transportation of d-glutamate.

## 6. Glutamate and NMDAR-Mediated Glutamatergic Signaling

Glutamate is the major excitatory neurotransmitter in the mammalian CNS. It is extensively distributed in the CNS, where it is almost exclusively located intracellularly. The NMDA receptor is a subgroup of iGluRs, selectively gated by specific agonists *N*-methyl-d-aspartate (NMDA) [15,64]. NMDAR is distinct from other iGluRs in its voltage-dependent activation via the removal of the Mg2+ blockade, high Ca^2+^ permeability, and relatively slow ligand-gated kinetics, rendering NMDAR unique and essential for its crucial role in synaptic function and plasticity [65,66]. At a resting membrane potential of about −70 mV, the Ca^2+^ channel of NMDAR is blocked by Mg^2+^. However, the strong and prolonged glutamate release during long-term potentiation (LTP) from the presynaptic terminal can activate α-amino-3-hydroxy-5-methyl-4-isoxazolepropionic acid receptors (AMPARs), resulting in depolarization and thereafter removing the Mg^2+^ blockade from the NMDAR channel and allowing the influx of Ca^2+^ ions. This strong activation of NMDARs leads to a Ca^2+^/calmodulin-dependent, protein kinase II (CaMKII)-mediated signaling cascade which enhances synaptic strength. In contrast, adequate NMDAR activation induces adequate increase in postsynaptic Ca^2+^ and leads to phosphatase-mediated, long-term depression (LTD) [67].

NMDARs play an important role in synaptic transmission and plasticity and seem critical for the survival of neurons by activating neuronal survival pathways [68,69]. Previous studies have reported that the blockade of NMDAR function causes neuronal apoptosis and degeneration [70,71]. These NMDAR-dependent neuroprotective functions mainly involve apoptosis inhibition and pro-survival transcription factors activation [72,73]. The activation of synaptic NMDARs may suppress the pro-apoptotic signaling pathways and molecules including caspases, apoptotic peptidase activating factor 1 (APAF1) and Puma in the cytoplasm. This may also promote the expression of the pro-survival transcriptional factors like cAMP responsive element binding protein (CREB) and inactivate the pro-death transcription factors like forkhead box protein O (FOXO) and p53. Finally, these effects may contribute to the inhibition of apoptosis and promotion of cell survival [72,73].

## 7. NMDAR-Mediated Glutamatergic Signaling in Alzheimer’s Disease

Inadequate synaptic NMDAR signaling may impair neuronal cell survival, while excessive NMDAR signaling may cause neurotoxicity. Abnormal glutamatergic signaling stimulation may damage or kill neurons [74]. Accumulating evidence has shown that glutamate excitotoxicity may be associated with delayed, slowly evolving neurodegeneration [75,76]. Increasing studies have shown that the toxicity is principally mediated by excessive Ca^2+^ entry, mainly via NMDARs [77,78,79,80], because NMDAR permeability is considerably higher for calcium ions than other iGluRs [81]. The adequate depolarization of the postsynaptic membrane and other factors that remove the Mg^2+^ blockade can mildly and chronically activate NMDARs, causing prolonged Ca2+ influx into the postsynaptic neuron. Excessive Ca^2+^ signaling stimulation results in the gradual loss of synaptic function and neuron death. This correlates with the progressive cognitive impairment and pathological neural anatomy development observed in AD patients. Based on these studies, the NMDAR antagonist memantine was developed as a neuroprotective treatment for AD [82,83,84]. Because NMDARs are important for cell survival, the level of NMDAR signaling must be maintained at a level sufficient to promote neuronal survival, yet not harmful to cause neurodegeneration as occurs in AD. Thus, the modulation of the NMDAR channel functions and glutamate availability are the critical factors influencing NMDAR signaling in AD.

The glutamate uptake and recycling system has an essential role in influencing the availability of glutamate-mediated neurotransmission. However, this system may be impaired in patients with AD. Previous studies have found that down-regulation of vesicular glutamate transporters are associated with abnormal amyloid precursor protein expression in AD patients [85,86,87]. Furthermore, it is reported that excitatory amino acid transporter 2 (EAAT2), primarily located in perisynaptic astrocytes, has impaired function in AD [88]. Studies using various species of Aβ peptides in neuronal cell culture seem to express the same notion that toxic Aβ may allow more glutamate availability by impairing glutamate uptake/recycling mechanisms [89,90,91]. This increased glutamate supply likely contributes to AD-associated excitotoxicity and neurodegeneration.

Glutamate availability may be affected by the presynaptic neurotransmitter release machinery. Aβ has been reported to potentially reduce significantly the expression of presynaptic proteins like synaptophysin, syntaxin, and synaptotagmin. Many of them are active components of the neurotransmitter release machinery [92]. A study indicated that endogenous Aβ plays a vital part in the regulation of activity-dependent synaptic vesicle release [93]. Deficits in the presynaptic vesicle release machinery supposedly compromise glutamate availability, making it less able to initiate an excitotoxicity effect. However, this is consistent with the pathological synaptic loss observed in AD and is likely a later effect that occurs in the ongoing neuronal degeneration.

AD is therefore associated with increased glutamate. Moreover, AD may enhance NMDAR signaling by modulating the receptor itself. Several studies showed that Aβ directly modulates the electrophysiological function of NMDARs. Generally, Aβ species elevate NMDAR-mediated synaptic currents and collateral toxicity. This can be either improved or blocked by NMDAR antagonists such as MK-801 [94,95,96], D-APV or memantine [97,98,99,100] that may prevent the structural effects of Aβ such as synaptic loss [101,102]. Aβ may directly or indirectly physically interact with NMDARs through synaptic proteins such as PSD95 [103,104,105].

## 8. Potential Role of d-glutamate in Alzheimer’s Disease

Pilot studies have reported decreased plasma d-glutamate levels are associated with cognitive impairment in AD [21,106]. Lin and colleagues enrolled 397 individuals and found d-glutamate level in patients with MCI, and AD was significantly lower than that of healthy controls (healthy elderly: 1620.4 ± 558.2, MCI: 1097.8 ± 284.0, mild AD: 1031.9 ± 775.8, moderate to severe AD: 598.3 ± 551.9) MMSE score was significantly associated with d-glutamate level (adjusted R square = 0.344) [106]. Another study comprising 144 patients (20 amnestic MCI, 85 mild AD, 25 moderate AD, and 14 severe AD patients) [21], which determined that the d-glutamate level was negatively correlated with the Alzheimer’s Disease Assessment Scale-Cognitive Subscale (ADAS-cog) behavior scores (r = −0.177, *p* = 0.034). These observations lead to the question: how is the lower peripheral d-glutamate level related to cognitive impairment? A pilot study enrolled eight individuals with MCI, nine individuals with AD, and 16 healthy elderly controls, finding that reduced hippocampal glutamate in MCI and AD was associated with episodic memory performance [107]. Another functional magnetic resonance imaging (fMRI) study included 15 patients with amnestic MCI and 22 age-, sex-, and education-matched healthy controls. A significant increase was observed in glutamate during a working memory task (both zero back and one back) in healthy controls, but no significant changes were detected in patients with MCI [108]. Mangas et al. observed that the d-glutamate level was higher in the cell body of the mouse brain regions, such as the ventral part of the mesencephalic central grey, the dorsal raphe nucleus, above the posterior commissure, the superior colliculus, and the subparafascicular thalamic nucleus [19]. Nevertheless, further studies are warranted to evaluate the relationship between cognitive impairment and the d-glutamate level in the brain tissue.

## 9. d-glutamate and Gut Microbiota

### 9.1. d-glutamate as a Component of Bacterial Cell Wall

d-glutamate is a component of the peptidoglycan cell wall in bacteria. In most Gram-negative bacteria, d-glutamate is generated via the glutamate racemase Murl [109]. Bacteria produce d-glutamate by a pyridoxal 5’-phosphate (PLP)-dependent glutamate racemase with tow cysteines involved in the catalysis [110,111]. Thereafter, bacteria like chlamydia use a UDP-*N*-acetylmuramoyl-l-alanine-d-glutamate ligase to add d-glutamate to the pentapeptide chain [112].

### 9.2. Glutamate Produced by Bacteria

Several bacterial strains can produce glutamate during food fermentation. For example, *Coryneform bacteria* are mainly used to produce glutamate in industry. LAB strains such as *Lactobacillus plantarum, Lactococcus lactis,* and *Lactobacillus paracasei* can synthesize glutamate [113,114]. A previous study has reported that approximately 15% of LAB strains can be detected in Asian fermented foods and produce glutamate [115]. From a functional point of view, glutathione-activated potassium channels were found only in the Synechocystis PCC 6803 strain, although over 100 prokaryotic channel proteins containing putative glutamate-binding domains have recently been identified [116]. Of these, 22 channels are homologs of the vertebrate iGlu receptor [117]. Furthermore, like eukaryotes, bacterial glutamate is a substrate for GABA synthesis by decarboxylation with glutamate decarboxylase, found in Gram-positive and Gram-negative bacteria [118,119]. These findings indicate glutamate produced by gut microbiota may modulate glutamate signaling [37].

### 9.3. Glutamate May be Modulated by Gut Microbiota

Pilot studies noted that the glutamate metabolized by gut microbiota may be associated with obesity [33], seizure [34], autism [35] and cognition [36]. A metagenome-wide association study showed that *Bacteroides thetaiotaomicron* was reduced in obese subjects and was inversely correlated with serum glutamate levels [33]. An animal study has revealed that hippocampal GABA/glutamate ratios can be modulated by the gut microbiota, affected by the ketogenic diet on epileptic seizures [34]. Wang et al. have reported that, in autism patients, the changes in gut microbiota are associated with the alterations in glutamate metabolism in the gastrointestinal tract. Moreover, they found that a lower abundance of two *Campylobacter jejuni* strains (81–176 and ICDCCJ07001) was associated with lower fumaric acid concentration in the guts of children with autism [35]. A previous study has shown that *Campylobacter jejuni* may activate glutamate synthesis [120]. Thus, lower abundance of *Campylobacter jejuni* may affect the synthesis of glutamate, which in turn would indirectly impact glutamate metabolism. Furthermore, a pilot study including 35 subjects showed that glutamate metabolized by gut microbiota is associated with cognitive functions such as processing speed and mental flexibility [36]. However, whether d-glutamate is modulated by gut microbiota in humans remains unclear.

### 9.4. Potential Role of d-glutamate in Brain-Gut-Microbiota Axis

l-glutamate is converted to d-glutamate by glutamate racemase. Non-pathogenic bacteria with glutamate racemase including *Corynebacterium glutamicum, Brevibacterium lactofermentum, Brevibacterium avium* [121], *Mycobacterium smegmatis* [122], and *Bacillus subtilis* [123] convert l-glutamate to d-glutamate. Among them, *Corynebacterium glutamicum* is commonly used in the food industry to produce glutamate [124]. It is believed to assist in conversion, but whether it does so in the gut microbiota in the human intestine remains to be confirmed. Prospective animal studies found that intestinal bacteria can regulate the metabolism of d-amino acids in the brain. The Kawase team noted that l-arginine(l-Arg), l-glutamine(l-Gln), l-isoleucine(l-Ile), and l-leucine(l-Leu) were significantly higher in specific pathogen-free (SPF) mice than in germ-free (GF) mice. However, d-Aspartate, d-serine, and l-serine were higher in some brain regions of GF mice than in those of SPF mice [20].

Pilot studies have found that decreased plasma d-glutamate levels are associated with cognitive impairment in AD [21,106]. Lin and colleagues reported that plasma d-glutamate level in patients with MCI and AD was significantly lower than that of healthy controls (healthy elderly: 1620.4 ± 558.2, MCI: 1097.8 ± 284.0, mild AD: 1031.9 ± 775.8, moderate to severe AD: 598.3 ± 551.9). MMSE score was significantly associated with d-glutamate level (adjusted R square = 0.344) [106]. Another study, comprising 144 patients (20 amnestic MCI, 85 mild AD, 25 moderate AD, and 14 severe AD patients) [21], noted that the d-glutamate level was negatively correlated ADAS-cog behavior scores (r = −0.177, *p* = 0.034).

Moreover, previous studies have found decreased glutamate in the brain is associated with impaired cognitive functions. A study of eight individuals with MCI, nine individuals with AD, and 16 healthy elderly controls found that reduced hippocampal glutamate in MCI and AD was associated with episodic memory performance [107], while another functional magnetic resonance imaging (fMRI) study noted reduced glutamate change in brain during a working memory task in patients with MCI [108].

Furthermore, benzoate, an inhibitor of d-amino acid oxidases (DAAO), can increase d-serine, thereby enhancing NMDAR activation [125,126]. Previous pilot studies have found that benzoate may improve cognition in schizophrenia patients [127,128]. Moreover, a pilot study including 60 patients showed that benzoate can improve cognition in patients with early-phase AD or MCI [5]. Based on these findings, we suggest that d-glutamate metabolized by gut microbiota may modulate the NMDAR-mediated glutamatergic signaling in AD patients.

## 10. Conclusions and Future Directions

Several studies have shown that the brain–gut–microbiota axis may significantly contribute to AD pathogenesis. Moreover, impaired memory and learning involve the dysfunctional signaling of glutamate, the major excitatory neurotransmitter in the brain. Gut microbiota and metabolite alterations including glutamate have been noted in neuropsychiatric disease patients. Gut microbiota including *Bacteroides vulgatus* and *Campylobacter jejuni* affect glutamate metabolism and decrease the glutamate metabolite, 2-keto-glutaramic acid. Furthermore, l-glutamate can be converted to d-glutamate with glutamate racemase and gut bacteria including *Corynebacterium glutamicum, Brevibacterium lactofermentum,* and *Brevibacterium avium*. Numerous pilot studies found that NMDAR-enhancing agents can improve cognitive functions in AD or Parkinson’s disease patients. These studies suggest that glutamate metabolized by gut bacteria may influence the glutamate NMDAR and cognitive functions in AD patients. Gut microbiota and d-glutamate may be potentially developed as novel intervention for dementia. Animal and human trials with glutamate-related NMDAR enhancers that evaluate extensive cognitive functions are suggested to evaluate the efficacy of glutamate signaling in gut microbiota in AD and other neurodegenerative dementia patients.

## Figures and Tables

**Table 1:** Distribution of d-glutamate in foods. The reported percentage of d-glutamate is relative to the total amino acid amounts.

Food		Relative Amount (%)	Analytical Method	Reference
**Coffee**				
	Roasted	32–41	gas chromatography	Palla et al. 1989 [39]
	Instant	27.4	gas chromatography	Brückner and Hausch 1989 [40]
	Green	< 0.2	gas chromatography	Palla et al. 1989 [39]
**Dairy/cheese**				
	Yakult	24.2	High Performance Liquid Chromatography	Jin et al. 1999 [41]
	Parmigiano Reggiano (24 months ripened)	15	gas chromatography	Marchelli et al. 2007 [42]
	Yogurt	12.4	gas chromatography	Brückner and Hausch 1990 [43]
	Emmentaler	6.2	gas chromatography	Brückner and Hausch 1989 [40]
	Kefir	4.9	gas chromatography	Brückner and Hausch 1989 [40]
	Sour milk	4.0	gas chromatography	Brückner and Hausch 1990 [43]
	Gorgonzola	1.5	gas chromatography	Brückner and Hausch 1989 [40]
**Fish**				
	Cooked pollock (95 °C)	16.5	High Performance Liquid Chromatography	Opstvedt et al. 1984 [129]
	Raw pollock	15.6	High Performance Liquid Chromatography	Opstvedt et al. 1984 [129]
	Cooked mackerel (95 °C)	14.7	High Performance Liquid Chromatography	Opstvedt et al. 1984 [129]
	Raw mackerel	12.9	High Performance Liquid Chromatography	Opstvedt et al. 1984 [129]
**Milk and milk powder**				
	Raw milk	2–3	gas chromatography	Palla et al. 1989 [39]
	UTH-milk	3–5	gas chromatography	Palla et al. 1989 [39]
	Infant formula	3–5	gas chromatography	Palla et al. 1989 [39]
	Milk powder	3–5	gas chromatography	Palla et al. 1989 [39]
**Vegetables**				
	Pickled cabbage	11.0	gas chromatography	Brückner and Westhauser 1994 [130]
	Garlic	0.5	gas chromatography	Brückner and Westhauser 1994 [130]
	Green cabbage	0.4	gas chromatography	Brückner and Westhauser 1994 [130]
	Red cabbage	0.3	gas chromatography	Brückner and Westhauser 1994 [130]
	Tomato	0.1	gas chromatography	Brückner and Westhauser 1994 [130]
**Vegetable juice**				
	Carrot	5.0	gas chromatography	Brückner and Hausch 1989 [40]
	Tomato	1.9	gas chromatography	Brückner and Westhauser 1994 [130]
	Red beet	1.0	gas chromatography	Brückner and Hausch 1989 [40]
	Celery	0.8	gas chromatography	Brückner and Hausch 1989 [40]
**Fruits**				
	Clementine	1.3	gas chromatography	Brückner and Westhauser 1994 [130]
	Orange	1.2	gas chromatography	Brückner and Westhauser 1994 [130]
	Lemon	1.1	gas chromatography	Brückner and Westhauser 1994 [130]
	Pear	0.9	gas chromatography	Brückner and Westhauser 1994 [130]
	Apple	0.5	gas chromatography	Brückner and Westhauser 1994 [130]
	Pineapple	0.4	gas chromatography	Brückner and Westhauser 1994 [130]
	Mango	0.4	gas chromatography	Brückner and Westhauser 1994 [130]
**Alcoholic beverages**				
	Wheat beer	16.2	gas chromatography	Brückner and Hausch 1989 [40]
	Red wine	3.0	gas chromatography	Brückner and Hausch 1989 [40]
	Beer	1.9	gas chromatography	Brückner and Hausch 1989 [40]
	Sake	1.1	gas chromatography	Brückner and Hausch 1989 [40]
	White wine	0.7	gas chromatography	Brückner and Hausch 1989 [40]
**Vinegar**				
	Balsamic	2.4	Ultra Performance Liquid Chromatography	Mutaguchi et al. 2013 [131]
	Apple	2	Ultra Performance Liquid Chromatography	Mutaguchi et al. 2013 [131]
	Tomato	0.2	Ultra Performance Liquid Chromatography	Mutaguchi et al. 2013 [131]
**Ham/meat**				
	Raw chicken	2.7	gas chromatography	Bunjapamai et al. 1982 [132]
**Other products**				
	Peanut butter	3.7	gas chromatography	Bunjapamai et al. 1982 [132]
	Liquid spice	3.0	gas chromatography	Brückner and Hausch 1989 [40]
